# Current Practices and Gaps in Integrating Point-of-Care Ultrasound in Neonatal and Pediatric Transport: A Scoping Review

**DOI:** 10.3390/diagnostics16030471

**Published:** 2026-02-03

**Authors:** Belinda Chan, Brighton Alvey, Brooke Barton, Yogen Singh

**Affiliations:** Division of Neonatology, Department of Pediatrics, University of California Davis Health, Sacramento, CA 95817, USA; belchan@health.ucdavis.edu (B.C.);

**Keywords:** POCUS, prehospital transport, interfacility transport, neonatal, pediatric

## Abstract

**Background:** Point-of-care ultrasound (POCUS) has emerged as a valuable tool for rapid diagnosis, procedural guidance, and real-time clinical decision-making in neonatal and pediatric critical care. Despite its growing use in acute medicine, the evidence describing its implementation, utility, and impact in interfacility and prehospital transport settings remains limited. This scoping review aims to systematically map the current body of evidence on POCUS use during neonatal and pediatric transport and to identify knowledge gaps to inform future research, training, and clinical integration. **Methods:** A scoping review was conducted following PRISMA-ScR 2020 guidelines, searching PubMed, Embase, Scopus, CINAHL, and Web of Science for studies describing POCUS use during neonatal and pediatric transport. **Results:** Of 3676 unique articles identified, 20 met inclusion criteria, including 10 cohort studies, 3 case series, 4 case reports, 2 narrative reviews, and 1 textbook chapter. Fifteen studies reported extractable patient-level data and were included in quantitative synthesis, encompassing 4278 patients. Among these, 1153 (27.0%) patients were under 18 years old, and 576 (13.5%) had POCUS performed during transport. POCUS was primarily used for diagnostic assessment—mainly lung and cardiac imaging—with variability in protocols, operator training, and transport characteristics. Eleven studies (73.3%) reported that POCUS altered clinical management, influencing management in 106 (18.4%) patients through diagnostic clarification, resuscitation decisions, medical or ventilator adjustments, and changes in transport destination. **Conclusions:** Evidence suggests that POCUS supports clinical decision-making and timely intervention during neonatal and pediatric transport, though use remains inconsistent. Future studies should focus on developing structured training frameworks, validating transport-specific protocols, and assessing the impact of POCUS on clinical outcomes and transport safety.

## 1. Background

Neonatal and pediatric transport plays a critical role in ensuring timely access to specialized care for critically ill infants and children. Each year, thousands of patients require interfacility or prehospital transport for advanced respiratory, cardiac, surgical, or extracorporeal support. These transports are often logistically complex and physiologically challenging. Referral hospitals involved in interfacility transport, as well as teams operating on scene during prehospital transfers, often have limited access to X-rays or other advanced diagnostic tools. During transport—whether by ground or air—clinicians face additional challenges, including confined spaces, poor lighting, and motion artifacts, which hinder accurate physical examination and monitoring. These limitations can delay recognition of deterioration or lead to suboptimal interventions.

Point-of-care ultrasound (POCUS) has become an important adjunct diagnostic and procedural tool in neonatal and pediatric critical care. It enables rapid, bedside evaluation of lung aeration, cardiac function, volume status, other hemodynamic parameters, vascular access guidance, and procedural safety [1,2,3,4]. Information obtained from POCUS can guide resuscitation and ongoing management. The portability and immediacy of POCUS make it particularly appealing in dynamic settings where timely decision-making is critical, such as during resource-limited transport settings.

In adult and prehospital medicine, POCUS is well established as a valuable adjunct for triage, diagnosis, and procedural support. Its use by emergency medical services and critical care transport teams has demonstrated improved diagnostic accuracy, expedited treatment decisions, and enhanced patient safety [5,6,7,8,9,10]. Applications include focused cardiac and lung assessments, detection of pneumothorax or pericardial effusion, confirmation of endotracheal tube (ETT) placement, and vascular access guidance—often leading to measurable improvements in survival and transport outcomes.

In contrast, the integration of POCUS into neonatal and pediatric transport remains in its early stages, despite its well-established use in inpatient critical care settings and adult transport. The transport environment poses unique challenges. The clinical applications of POCUS during adult transport differ substantially from those relevant to neonatal and pediatric patients. The existing literature on transport POCUS is sparse. Consequently, significant evidence gaps persist regarding its feasibility, diagnostic accuracy, clinical impact, and effects on transport efficiency and safety in the pediatric population. This scoping review aims to systematically map the current body of evidence on POCUS use during neonatal and pediatric transport and to identify knowledge gaps to inform future research, training, and clinical integration.

## 2. Methods

### 2.1. Study Design

A scoping review was conducted according to the PRISMA-ScR (Preferred Reporting Items for Systematic Reviews and Meta-Analyses extension for Scoping Reviews) 2020 guidelines [11].

### 2.2. Search Strategy

The search was performed through 5 databases, including PubMed, Embase, Scopus, CINAHL, and Web of Science, from inception through September 15, 2025. Keywords included: “ultrasound,” “POCUS,” “sonography,” “neonatal,” “neonate,” “newborn,” “infant,” “pediatric,” “child,” “prehospital,” “emergency medical services.” Additionally, the reference lists of relevant articles and reviews were searched for additional articles. No restrictions on publication date were applied. See Supplementary Materials (Files S1–S3) for the full search strategy.

Eligible studies were full-text articles, including case reports, case series, retrospective reviews, observational studies, guidelines, narrative reviews, and technical reports that described prehospital or interfacility transport practices involving the use of POCUS for diagnostic or procedural purposes in neonates and pediatric patients younger than 18 years. Studies were excluded if they focused exclusively on adult populations, did not explicitly stratify data by age or pediatric subgroups, or used POCUS only in non-transport settings. Non-English language studies without available translation were also excluded.

During the initial screening phase, a single reviewer assessed titles and abstracts for potential inclusion. To assess screening reliability, a predefined subset of 736 of 3676 records (20.0%) was independently screened by a second reviewer. Inter-rater reliability was calculated using Cohen’s kappa. The two reviewers demonstrated 81.7% raw agreement and moderate reliability after adjusting for chance (κ = 0.52). Discrepancies identified within the dual-screened subset were resolved through discussion and consensus. All studies meeting the preliminary inclusion criteria subsequently underwent full-text review, conducted independently by two reviewers; disagreements were resolved by consensus prior to data extraction.

### 2.3. Data Extraction and Synthesis

The studies were stored in the citation manager software EndNote (Clarivate Analytics, Philadelphia, PA, USA), and the two reviewers abstracted data using a standardized data abstraction form. Abstracted data captured key study characteristics, including author name, publication year, study design, country of origin, prehospital clinician and ultrasound operator, patient population, indication for transport, indication for ultrasound, and study performed. The specific details of ultrasound implementation were analyzed through full-text review and grouped into categories based on the study indication (diagnostic or interventional), the study type performed, and outcomes during ultrasound-guided procedures or after diagnostic ultrasound acquisition. The transport context in each study was categorized as ground-based or air medical, and prehospital or interfacility, as indicated in the text. The indication for transport was documented. Of the studies that reported limitations in ultrasound acquisition or utility, limitations were categorized into the following groups: technological limitations, knowledge or training gaps, and transport-related issues. Two reviewers independently abstracted data from all included studies, with discrepancies resolved by consensus or a third reviewer as needed per PRISMA-ScR guidelines.

### 2.4. Data Analysis

Descriptive statistics were used to characterize POCUS implementation during transport practices in each included study. Quantitative data (e.g., study counts, frequencies of POCUS applications, reported outcomes) were summarized using descriptive statistics and presented in tables and figures to map the breadth and distribution of evidence.

Qualitative data (e.g., narrative descriptions of implementation barriers, facilitators, and clinical impact) were analyzed using thematic analysis. An inductive approach was used, allowing themes to emerge directly from the data rather than applying a predefined theoretical framework. Full-text articles were reviewed, and qualitative data were coded iteratively to identify recurring concepts. Codes were grouped into higher-order themes through reviewer consensus and refined through cross-study comparison, yielding overarching domains related to POCUS implementation, clinical utility, and limitations.

## 3. Results

### 3.1. Study Selection

**Figure 1:**
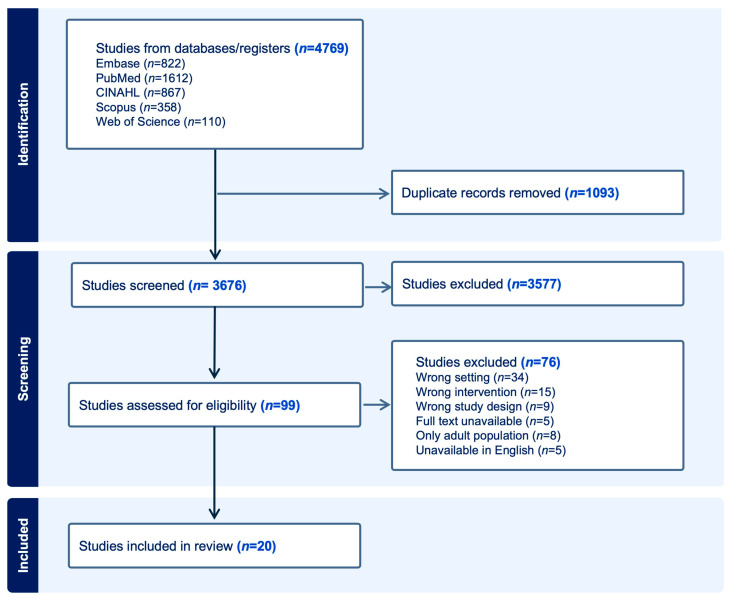
PRISMA flow diagram summarizing the inclusion of studies.

A total of 4769 records were found through database searches. After removing 1093 duplicates, 3676 records remained for title and abstract screening. Of these, 3577 records were excluded based on the predefined eligibility criteria. The full texts of 99 articles were then evaluated for eligibility. Ultimately, 20 studies met the inclusion criteria and were included in the final review (Figure 1).

### 3.2. Study Characteristics

The final review included 20 studies: 10 large cohort studies (2 retrospective, 8 prospective), 3 case series, 4 case reports, 2 narrative reviews, and 1 textbook chapter. Of these, 15 studies reported original data on pediatric or neonatal patients who underwent ultrasound during transport and were therefore included in the quantitative results. The remaining five sources (two narrative reviews, one textbook chapter, and two medical education articles) discussed POCUS in pediatric transport conceptually but did not provide extractable patient-level data—these were incorporated qualitatively to contextualize findings but were not included in numeric analyses. Studies with extractable patient-level data encompassed 4278 patients. Of these, 1153 (27.0%) were pediatric or neonatal patients under 18 years. 576 of the 1153 patients (50.0%) met the full inclusion criteria as pediatric or neonatal patients who underwent POCUS during transport. Study focuses were distributed as follows: three examined clinical outcomes, ten were descriptive, five addressed feasibility or pilot testing, and two focused on training or education (Table 1).

Age reporting across included studies was highly heterogeneous and inconsistently structured, with data presented as individual ages, summary statistics (mean, median, range), or counts within specific age categories. Five studies included neonatal-only cohorts, with ages ranging from minutes after birth to a median of a few hours [12,13,14,15]. Two studies covered both pediatric and neonatal cohorts, reporting broad age distributions, including Campos et al., with a median age of 85 days, and Becerra-Hervás et al., with ages ranging from 14.9 months to 81.5 months [16,17]. Several studies reported single pediatric cases aged 7–17 years [18,19,20,21,22,23]. Larger studies provided age categories, spanning infancy through adolescence [24,25]. Overall, age was reported inconsistently using individual ages, means, medians, or grouped categories (Supplementary material File S4).

### 3.3. POCUS Implementation

Among 1153 pediatric and neonatal patients transported across the included studies, 576 (50.0%) had POCUS evaluation during transport. Indications for transport were most commonly trauma (309 of 1153 patients, 26.8%) and respiratory distress (255 of 1153, 22.1%), although nearly half (559 of 1153, 48.5%) did not report a specific indication. Ground transport accounted for 152 of 1153 cases (13.2%), air medical transport for 4 of 1153 (<1%), and transport type was not reported for 997 of 1153 cases (86.5%). Most transports occurred in prehospital settings (656 of 1153, 56.9%), followed by interfacility transfers (298 of 1153, 25.8%) (Table 2).

POCUS was used almost exclusively for diagnostic purposes (575 of 576 scans, 99.8%). Lung ultrasound was the most frequently reported acquisition type (215 of 576, 37.3%), followed by cardiac ultrasound (33 of 576, 5.7%). Multiple anatomic regions were scanned in 83 patients (14.4%), and acquisition type was not reported for 243 patients (42.2%). Carmo et al. reported 4 patients who underwent cardiac ultrasound, with 3 also undergoing head ultrasound [13]. Byhahn et al. described a single patient who underwent both cardiac and abdominal imaging [20]. Campos et al. reported 23 patients who received lung, cardiac, and head imaging, while Browning-Carmo et al. reported 55 patients who underwent cardiac, abdominal, and head ultrasounds [15,17]. Reid et al. described one patient who received a procedural ultrasound for a fascial iliac block [19] (Table 2).

Ultrasound equipment type was documented for 332 patients; 130 (22.6%) underwent handheld ultrasound examinations, and 202 (35.1%) were scanned using portable cart-based or laptop systems. Equipment type was not reported for 244 patients (42.4%). Table 2 provides a detailed summary of these findings.

### 3.4. Use of Established Protocols

Across the 15 studies with extractable patient-level data, 7 (46.7%) reported using a protocolized approach to POCUS use during transport, while 8 (53.3%) did not specify a protocol. The most frequently reported standardized approach was protocolized lung ultrasound [12,14]. Focused Assessment with Sonography in Trauma (FAST) examinations were reported in three studies, including a large cohort from Karfunkle (*n* = 168) and single-patient scans in two case reports [20,21,25]. Focused Echocardiogram Evaluation for Life Support (FEEL) was used in two single-patient reports [20,22]. A single study, Becerra-Hervás et al., employed the widest array of protocolized imaging, reporting the use of protocolized lung ultrasound (LUS), Bedside Lung Ultrasound in Emergency (BLUE), Rapid Ultrasound for Shock and Hypotension (RUSH), extended-FAST (eFAST), and Cardiac Arrest Ultrasound Exam (CAUSE) protocols in all 89 patients, with each patient undergoing all listed protocol components [16]. Table 1 provides a detailed summary of protocol use across studies.

Across the studies, ultrasound examinations were predominantly performed by physicians, accounting for 408 of 576 pediatric scans (70.8%). All physician-operators received formal ultrasound training. However, one large study reported operator type across 3291 ultrasound examinations performed across all age groups, without stratification by pediatric subset [25]. In this mixed-age cohort, paramedics performed 2486 ultrasounds (75.6%), Emergency Medical Technicians (EMT) performed 72 (2.2%), registered nurses 61 (1.9%), and physicians 57 (1.7%). The operator was not documented in 631 examinations (19.2%). Because the operator type was not age-stratified in this large cohort, these findings cannot be assumed to represent pediatric-specific practice patterns and should be interpreted with caution when drawing conclusions about operator type in neonatal and pediatric transport. Additionally, this study did not explicitly report the level or type of ultrasound training received by operators. The heterogeneity in operator reporting and the inclusion of non-stratified data limit definitive conclusions about optimal operator characteristics for pediatric transport POCUS. (Table 2)

### 3.5. Impact on Clinical Decision-Making

Across the 15 studies with extractable data, 11 studies (73.3%) reported that POCUS use during transport contributed to at least one change in clinical management or decision-making. Among the 576 transported patients who underwent ultrasound, 106 (18.4%) experienced at least one POCUS-related change in management, totaling 116 discrete management change events (Table 3). The frequency of change varied by study, ranging from isolated single-patient decisions in case reports to larger cohorts in which multiple patients underwent POCUS-directed management adjustments.

Management-change events were heterogeneous and are summarized in Table 4. The most frequently reported category was diagnostic clarification, accounting for 36 events (31.0%), followed by changes in medical management (26 events, 22.4%) and ventilator adjustments (24 events, 20.7%). Additional impacts included transport destination changes (14 events, 12.1%), procedural interventions such as chest tube placement or pericardial drainage (9 events, 7.8%), airway management changes (6 events, 5.2%), and resuscitation-related decisions (1 event, <1%). Because individual patients could undergo more than one POCUS-related intervention, totals reflect discrete events rather than unique patients. Supplemental material (File S5) details extracted data on changes in management across the included studies. No studies reported any patient safety issues or adverse events related to ultrasound imaging during transport.

### 3.6. Barriers and Challenges

5 of the 15 studies (33.3%) explicitly reported barriers to POCUS implementation during pediatric or neonatal transport. Reported barriers include a lack of training [19,20,21], extra time spent on scanning [26], the added weight of the scanner to the flight planning, an extra trolley to set up the ultrasound laptop, scanning may influence patients’ stabilization, and difficult to gain adequate ultrasound windows when scanning from an unconventional position during transport [20]. See Supplementary Materials (File S6) for more details on reported barriers and challenges.

### 3.7. Training and Educational Sources

Leviter et al. evaluated a remote learning and teleguidance curriculum for pediatric critical care transport teams to use POCUS to confirm ETT placement. There are multiple methods to confirm ETT placement. In this study, two ultrasound approaches were used: (1) direct visualization of the trachea and esophagus with a probe placed over the anterior neck, where esophageal intubation is suggested by air artifact in the normally collapsed esophagus (the “double trachea” sign); and (2) indirect confirmation by assessing bilateral lung sliding, which indicates effective ventilation. Operators were pediatric transport clinicians. The study implemented a structured remote training program, including didactic modules and real-time teleguidance during patient transport. Outcomes demonstrated that transport clinicians could reliably acquire and interpret airway ultrasound images to confirm ETT placement, with high concordance to standard confirmation methods. The curriculum improved operator confidence and skill, supporting the feasibility of remote POCUS education for airway management in pediatric transport settings. Specific values and percentages are not provided in the published literature [26].

Stritzke et al. investigated the use of POCUS by neonatal transport clinicians to evaluate cardiac function during neonatal transport. The study focused on the feasibility and accuracy of bedside cardiac ultrasound performed by transport team members. Operators were neonatal transport clinicians trained in basic cardiac POCUS. The primary outcomes were the ability to obtain interpretable cardiac images and assess global cardiac function in neonates during transport. The study found that transport clinicians could successfully perform and interpret cardiac POCUS, providing real-time hemodynamic information that influenced clinical management. Specifics included high rates of image acquisition and interpretation, with POCUS findings leading to changes in patient care during transport [27].

### 3.8. Qualitative Studies

Job and colleagues provide a focused overview of POCUS applications for infants and children with shock during transport. The authors emphasize that multiorgan ultrasound—particularly cardiac, lung, and abdominal views—supports rapid differentiation of shock etiologies and enables timely, targeted intervention during transfer. The chapter highlights the utility of structured protocols, systematic image acquisition, and ongoing reassessment, underscoring the role of POCUS as a real-time decision-support tool in resource-constrained or time-critical transport environments [28].

Foster et al. present a narrative review describing the expanding use of POCUS in pediatric and neonatal emergency transport, particularly for evaluating respiratory compromise, hemodynamic instability, and procedural guidance. Their review underscores the potential of POCUS to expedite diagnosis, enhance situational awareness, and improve clinical safety during transport. The authors advocate for integrating POCUS into transport protocols and training pathways to ensure consistent, effective use across transport teams [29].

In another comprehensive review, Carmo et al. outline the historical development of neonatal transport ultrasound, tracing the evolution of POCUS from a specialized imaging modality to a portable, bedside tool accessible to transport clinicians. The paper emphasizes the importance of structured training, competency assessment, and institutional support for successful implementation. The historical perspective reinforces the need for standardized protocols, robust quality assurance processes, and sustained investment in training to ensure safe and effective POCUS use in neonatal transport, consistent with emerging guidelines and expert consensus [30].

## 4. Discussion

This scoping review is the first to examine POCUS use in neonatal and pediatric transport. Findings explicitly show that POCUS is feasible and influences clinical decision-making in about 18% of reported cases. It serves as a valuable extension of the physical examination in time-critical, resource-limited transport settings where conventional imaging is unavailable. However, implementation challenges persist. Mapping current evidence and practices provides a foundation for developing standardized protocols, training, and quality frameworks to support safe and effective integration of POCUS into neonatal and pediatric transport medicine.

Our findings align with adult transport literature, where POCUS has shown to be feasible and beneficial [5,6,7,8,9,10]. But variability in operator training, absence of standardized protocols, and inconsistent quality assurance processes have hindered widespread adoption in the adult transport system [5,6,7,8,9,10].

Adult transport POCUS studies help inform feasibility, workflow, and real-time decision-making during transport. However, findings from adult populations cannot be directly applied to neonatal and pediatric patients due to differences in anatomy, disease processes, transport equipment, and treatment priorities. While the use of ultrasound in confined transport settings, short focused examinations, and some common POCUS-guided diagnoses are generalizable across age groups, scanning protocols, image acquisition techniques, scope of practice, and physiologic interpretation would be age specific. Therefore, adult transport studies are included in this review to inform implementation principles rather than pediatric-specific clinical recommendations.

**Figure 2:**
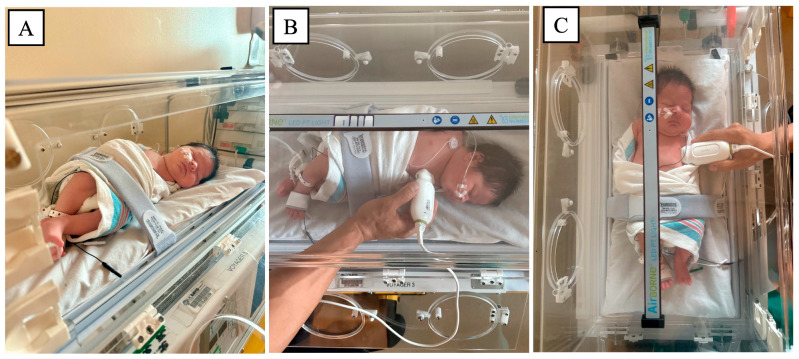
Proposed positioning and probe placement techniques for neonatal point-of-care ultrasound (POCUS) performed within an incubator. (**A**) Left side-lying positioning with low swaddle and low belt to optimize POCUS acquisition. (**B**) Cardiac probe placement in the left parasternal position within the incubator. (**C**) Lung ultrasound probe placement for anterior lung views while maintaining incubator constraints.

In response to these gaps, and in the absence of neonate-specific transport frameworks, we propose several practice-informed strategies to facilitate POCUS integration in neonatal and pediatric transport. These proposals are intended to be hypothesis-generating and to inform future prospective studies and protocol validation efforts. One key barrier is the physical constraint of the transport environment itself. Cardiac and lung ultrasound acquisition during transport is limited by restricted space within incubators and requires safety restraints. Lacking supporting literature guidance, we propose a neonatal positioning approach based on our clinical experience. The height and length of the incubator limit probe maneuverability, preventing the ultrasound transducers from being positioned upright or in a conventional scanning orientation. We therefore suggest a left side–lying position with a low swaddle and low belt to facilitate cardiac and lung POCUS acquisition (Figure 2), while maintaining the rapid, focused POCUS required during transport.

Although positioning may facilitate image acquisition, effective transport POCUS also requires a structured, time-limited approach targeting actionable diagnoses. In adult trauma transport, eFAST remains appropriate and well established. However, in non-trauma neonatal and pediatric transport, deterioration more often reflects cardiopulmonary instability (e.g., respiratory failure, myocardial dysfunction, pulmonary hypertension, or pericardial effusion) rather than hemorrhagic injury, and trauma-focused protocols may not address the most relevant diagnostic targets.

**Figure 3:**
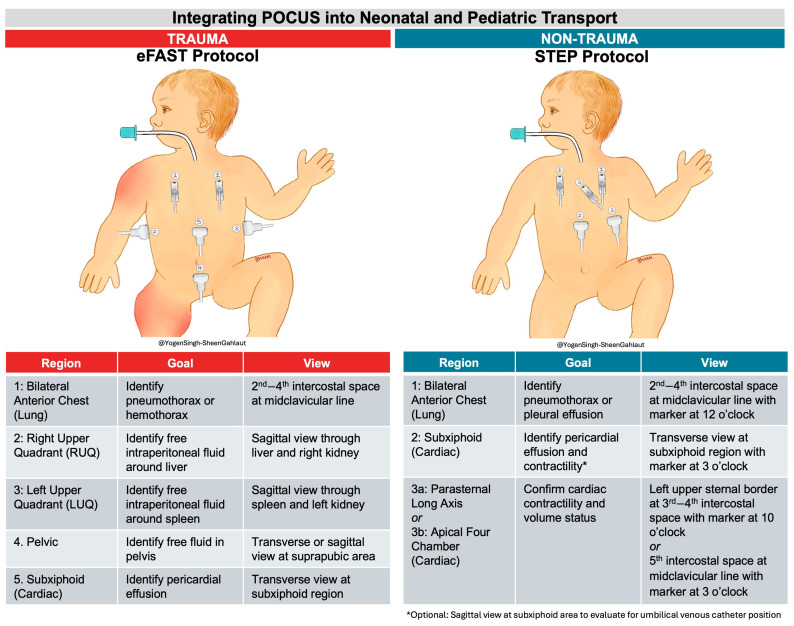
Neonatal and pediatric transport protocols, including eFAST (extended Focused Assessment with Sonography in Trauma) and our proposed protocol, STEP (Structured Transport Emergency POCUS), based on clinical indications.

Across the studies in this review, lung and cardiac ultrasound were the most frequently used and clinically impactful applications in neonate and pediatric population, yet no transport-specific framework exists to guide their focused use in medical deterioration. To address this gap, we propose the Structured Transport Emergency POCUS (STEP) framework as a conceptual, hypothesis-generating approach for rapid lung and cardiac assessment during non-trauma neonatal and pediatric transport. (Figure 3) STEP is intended to inform future protocol development and testing rather than serve as a validated or practice-ready recommendation. STEP framework consists of focused lung and cardiac assessments—lung ultrasound for pneumothorax and pleural effusion requiring immediate intervention, and cardiac ultrasound for contractility, volume status, and pericardial effusion to guide real-time hemodynamic management. Figure 3 illustrates the specific scanning goals and the corresponding ultrasound views to be obtained. (Figure 3) Other ultrasound applications—such as endotracheal tube (ETT) confirmation, umbilical venous catheter positioning, and lung pathology assessment—may offer additional value; however, they can extend scan duration and potentially delay transport. Therefore, these applications are considered optional in our proposed protocol. The STEP protocol is designed to balance diagnostic utility with efficiency, supporting timely stabilization and safe transfer of critically ill infants and children.

Ultrasound equipment was identified as a key barrier to implementation. Table 5 outlines essential considerations for selecting transport-appropriate machines.

Addressing the training gap is equally critical. Training programs should be developed that focus on the specific transport scanning protocols proposed and align with common transport indications. Stritzke et al. suggest that with appropriate training, transport providers can reliably acquire and interpret transport-specific cardiac ultrasound images to inform real-time management [27]. Remote learning and teleguidance, as demonstrated by Leviter et al., offer promising strategies for expanding POCUS education to transport teams in diverse geographic and resource settings [26].

The impact of POCUS on clinical outcomes during neonatal and pediatric transport remains poorly defined. While existing studies describe management changes, no trials have evaluated patient-centered outcomes such as mortality, length of stay, or complication rates. Future research in neonatal and pediatric transport POCUS should prioritize prospective, multicenter studies designed to evaluate patient-centered outcomes while accounting for the unique constraints of the transport environment. Given the low-frequency and high-acuity nature of transport settings, outcomes such as mortality alone may be insufficiently sensitive. Instead, studies should prioritize safety outcomes (e.g., adverse events or delays during transport), communication and decision-making measures (e.g., diagnostic confidence, escalation of care, or destination changes), and clinical effectiveness metrics (e.g., time to diagnosis, ultrasound-guided procedural success rate, or other ultrasound-directed management changes). Additional priorities include evaluating training, competency, and quality assurance frameworks specific to neonatal and pediatric transport teams.

## 5. Limitations

We acknowledge several limitations of this scoping review. First, small sample sizes in neonatal and pediatric populations within the broader prehospital ultrasound literature limit the strength and generalizability of findings, underscoring the scarcity of pediatric-specific research. Second, as typical of scoping reviews, included studies varied considerably in design, methodological rigor, and reporting quality. Third, substantial heterogeneity in ultrasound protocols, equipment, operator expertise, and outcome measures precluded quantitative synthesis and definitive conclusions about the clinical impact of transport POCUS. This variability highlights the need for standardized reporting frameworks tailored to transport medicine. Fourth, a significant limitation is the inclusion of non-age-stratified data from mixed-cohort studies. Fifth, interpretation of certain findings is limited by incomplete reporting across studies, including frequently not reported data for variables such as mode of transport and image acquisition type, highlighting the need for standardized reporting in the transport realm. Next, exclusion of non-English publications and gray literature may have limited representation of transport POCUS practices from non-English-speaking regions and unpublished programs. Finally, most studies involved physician-performed scans, limiting the applicability of results to transport teams with diverse provider compositions and scopes of practice.

## 6. Conclusions

This scoping review demonstrates that POCUS is both feasible and clinically valuable during neonatal and pediatric transport, and may improve clinical decision-making and patient management. POCUS is a powerful clinical adjunct, especially in limited-resource settings, such as during transport, where specialist expertise and other diagnostic modalities are limited. However, significant challenges remain in its widespread implementation. Future studies should focus on standardizing transport-specific POCUS protocols, developing consensus-based training curricula and credentialing frameworks, and rigorously evaluating their impact on clinical outcomes to define their role within neonatal and pediatric transport medicine.

## Figures and Tables

**Table 1:** Study characteristics. Summary of the literature on pediatric ultrasound use during transport. Abbreviations: BLUE, bedside lung ultrasound in emergency; CAUSE, cardiac arrest ultrasound examination; FAST, focused assessment with sonography for trauma; FEEL, focused echocardiography in emergency life support; LUS, lung ultrasound; NA, not applicable; RUSH, rapid ultrasound for shock and hypotension; Pts, patients; Yr, years., # number.

Reference #	First Author	Yr	Study Design	# of Pts (*n*)	# of Pediatric Pts (*n*, %)	Pediatric Pts Who Underwent Ultrasound (*n*, %)	Study Focus	Scan Protocol Used
[12]	Jagla	2019	Prospective study	50	50 (100%)	50 (100%)	Clinical outcomes	LUS: 50
[13]	Carmo	2013	Case series	4	4 (100%)	4 (100%)	Feasibility or Pilot	
[14]	Ollier	2022	Prospective study	76	76 (100%)	76 (100%)	Feasibility or Pilot	
[15]	Browning-Carmo	2017	Prospective study	55	55 (100%)	55 (100%)	Feasibility or Pilot	
[16]	Becerra-Hervás	2025	Prospective study	89	89 (100%)	89 (100%)	Clinical outcomes	LUS: 89 BLUE: 89 RUSH: 89 eFAST: 89 CAUSE: 89
[17]	Campos	2021	Prospective study	23	23 (100%)	23 (100%)	Descriptive	
[18]	Mommers	2023	Case series	2	2 (100%)	1 (50%)	Feasibility or Pilot	
[19]	Reid	2023	Case report	1	1 (100%)	1 (100%)	Descriptive	
[20]	Byhahn	2008	Case report	1	1 (100%)	1 (100%)	Descriptive	FAST: 1 FEEL: 1
[21]	Campo dell’ Orto	2014	Case report	1	1 (100%)	1 (100%)	Descriptive	FAST: 1 FEEL: 1
[22]	Steiger	2009	Case report	1	1 (100%)	1 (100%)	Descriptive	FEEL: 1
[23]	Polk	2004	Case series	3	1 (33.3%)	1 (33%)	Descriptive	
[24]	Nielsen	2022	Retrospective study	651	651 (100%)	75 (11.5%)	Clinical outcomes	
[25]	Karfunkle	2024	Retrospective study	3291	168 (5.1%)	168 (5.1%)	Descriptive	FAST: 168
[26]	Leviter	2022	Prospective study	0	NA	NA	Training or Educational	
[27]	Stritzke	2019	Prospective study	0	NA	NA	Training or Educational	
[28]	Job	2023	Narrative review	0	NA	NA	Descriptive	
[29]	Foster	2021	Narrative review	0	NA	NA	Descriptive	
[30]	Browning-Carmo	2017	Narrative review	0	NA	NA	Descriptive	
[31]	Boet	2017	Prospective study	30	30 (100%)	30 (100%)	Feasibility or Pilot	
Totals				4278	1153 (27.0%)	576 (13.5%)		

**Table 2:** Summary of point-of-care ultrasound (POCUS) implementation during pediatric and neonatal transport. Abbreviations: POCUS, point-of-care ultrasound.

	*n* (%)
Total neonates and pediatric patients who underwent transport	1153
Total neonates and pediatric patients who underwent transport and had an ultrasound performed	576
Indication for transport, *n* = 1153	
Cardiac arrest	24 (2.1%)
Trauma	309 (26.8%)
Respiratory distress	255 (22.1%)
Obstetric complication	1 (0.1%)
Need for invasive procedure	5 (0.4%)
unavailable at departure site	
Not reported	559 (48.5%)
Transport type, *n* = 1153	
Ground transport	152 (13.2%)
Air medical	4 (0.3%)
Not reported	997 (86.5%)
Transport context, *n* = 1153	
Prehospital transport	656 (56.9%)
Interfacility transfer	298 (25.8%)
Not reported	199 (17.3%)
POCUS indication, *n* = 576	
Diagnostic	575 (99.8%)
Procedural	1 (0.1%)
Ultrasound acquisition, *n* = 576	
Cardiac	33 (5.7%)
Lung	215 (37.3%)
Abdomen	1 (0.1%)
Head	0 (0%)
Procedural (nerve block)	1 (0.1%)
Multiple (Cardiac, lung, abdomen, head, and/or vessel)	83 (14.4%)
Not reported	243 (42.2%)
**Ultrasound equipment type, ***n* = 576	
Handheld	130 (22.6%)
Portable cart or laptop	202 (35.1%)
Not reported	244 (42.4%)

**Table 3:** Summary of reported changes in clinical management attributable to point-of-care ultrasound (POCUS) during neonatal and pediatric transport. One patient may have had more than one POCUS-related management change. Therefore, subtype counts do not sum to the overall number of patients who experienced a change in management, # number.

Reference #	Reported Change in Management?	Number of Patients Who Underwent Change in Management, *n* (%)	Number of Change-in Management Events, *n*
[26]	No	0/75	0
[23]	No	0/168	0
[25]	Yes	1/1 (100%)	1
[15]	Yes	1/1 (100%)	3
[16]	Yes	1/1 (100%)	2
[30]	Yes	1/1 (100%)	1
[28]	Yes	1/1 (100%)	1
[13]	No	0/30	0
[29]	Yes	1/1 (100%)	1
[18]	Yes	4/4 (100%)	10
[21]	Yes	21/50 (42.0%)	21
[17]	Yes	14/23 (54.0%)	14
[27]	No	0/76	0
[14]	Yes	29/55 (52.7%)	30
[12]	Yes	32/89 (35.9%)	32
Totals	11/15 (73.3%)	106/576 (18.4%)	116

**Table 4:** Categories and frequencies of POCUS-associated management changes during neonatal and pediatric transport.

Type of Change in Management	Number of Events, *n* (%)	Description
Diagnostic clarification	36 (31.0%)	Refined diagnosis, altered differential
Resuscitation-related decisions	1 (0.9%)	Guided resuscitation, shock assessment, cardiac arrest
Changes in medical management	26 (22.4%)	Adjusted fluids, inotropes, vasoactive medications, surfactant, analgesia
Ventilator adjustments	24 (20.7%)	Modified ventilator settings based on POCUS findings
Procedural interventions	9 (7.8%)	Chest tube placement, pericardial drainage
Airway management changes	6 (5.2%)	POCUS-guided ETT repositioning
Transport destination changes	14 (12.1%)	Redirected to higher/lower acuity center
Total management change events	116 (100%) *	* Because a single patient may experience multiple POCUS-associated management changes, totals represent discrete events rather than unique patients.

**Table 5:** Key Considerations for Selecting and Using a POCUS Machine in Neonatal and Pediatric Transport.

Feature Category	Key Considerations	Relevance to Transport Use
Portability and design	Compact, lightweight system Long battery life (≥2–4 h) Durable, shock- and vibration-resistant Mountable within transport vehicles Hand-held for in referral hospital or at scene	Ensures usability in confined spaces (ambulance, aircraft); withstands vibration and movement during transport
Image quality and performance	High-resolution imaging (up to 12–15 MHz) Rapid boot-up and minimal lag	Enables quick, reliable imaging in time-critical settings
Probes and applications	Linear (lung, vascular), Phased array (cardiac), Curvilinear (abdominal) Small-footprint probes for neonates Tangle-free or retractable cables	Provides flexibility for multi-organ scanning even for small patients. A single phased-array probe may be sufficient for most transport applications and can reduce the number of equipment required.
Usability and interface	Simple touchscreen or hybrid interface One-handed operation Scan presets (lung, cardiac, eFAST) Screen is readable in both bright sunlight and low-light conditions.	Facilitates rapid assessment and reduces cognitive load during critical interventions.
Connectivity and data management	Wi-Fi or cellular data transfer DICOM and PACS compatibility HIPAA-compliant cloud storage	Allows real-time image sharing with specialists at receiving centers, supporting tele-ultrasound. Because Wi-Fi may be unreliable in remote settings or transits, devices should not rely on wireless connectivity for imaging or data transfer.
Cleaning and infection control	Compatible with standard disinfectants Disposable probe covers	Supports infection control between patient use.
Training and integration	Transport-specific training modules Quality assurance via image review Defined scanning roles within team	Promotes standardized performance and integration of POCUS into routine transport protocols

## Data Availability

All data included in this review are derived from published studies. Extracted data and Supplementary Materials are available from the corresponding author upon reasonable request.

## Supplementary Materials

The following supporting information can be downloaded at: https://www.mdpi.com/article/10.3390/diagnostics16030471/s1, File S1: Preferred Reporting Items for Systematic reviews and Meta-Analyses extension for Scoping Reviews (PRISMA-ScR) Checklist; File S2: Search Criteria; File S3: Standardized data extraction form; File S4: Summary of age reporting across included studies. Pediatric age groups include patients aged 1 month to 17 years. Neonatal groups include patients less than 1 month old. The “all ages” group includes neonatal, pediatric, and adult patient populations. File S5: Extracted data of reported changes in clinical management or decision-making attributable to POCUS during neonatal and pediatric transport across 17 included studies. File S6: Reported barriers and challenges to POCUS implementation during pediatric and neonatal transport among 15 studies with extractable patient-level data, categorized by barrier type and subtype. Note: One patient may have had more than one POCUS-related management change. Therefore, subtype counts do not sum to the overall number of patients who experienced a change in management.

## References

[1] Stewart D.L., Elsayed Y., Fraga M.V., Coley B.D., Annam A., Milla S.S. (2022). Use of Point-of-Care Ultrasonography in the NICU for Diagnostic and Procedural Purposes. Pediatrics.

[2] Conlon T.W., Baker D., Bhombal S. (2024). Cardiac point-of-care ultrasound: Practical integration in the pediatric and neonatal intensive care settings. Eur. J. Pediatr..

[3] Lee M.S., Sweetnam-Holmes D., Soffer G.P., Harel-Sterling M. (2024). Updates on the clinical integration of point-of-care ultrasound in pediatric emergency medicine. Curr. Opin. Pediatr..

[4] Yousef N., Singh Y., De Luca D. (2022). “Playing it SAFE in the NICU” SAFE-R: A targeted diagnostic ultrasound protocol for the suddenly decompensating infant in the NICU. Eur. J. Pediatr..

[5] Hellenthal K.E.M., Porschen C., Wnent J., Lange M. (2025). Evolving role of point-of-care ultrasound in prehospital emergency care: A narrative review. Scand. J. Trauma. Resusc. Emerg. Med..

[6] Amaral C.B., Ralston D.C., Becker T.K. (2020). Prehospital point-of-care ultrasound: A transformative technology. SAGE Open Med..

[7] Shekhar A.C., Blumen I. (2021). A narrative review on the use of ultrasonography in critical care transport: Is POCUS hocus?. Trends Anaesth. Crit. Care.

[8] Warren J., Tamhankar O., Toy J., Schlesinger S., Liu Y. (2024). The Use of Prehospital Ultrasound: A Scoping Review. Ann. Emerg. Med..

[9] von Foerster N., Radomski M.A., Martin-Gill C. (2024). Prehospital Ultrasound: A Narrative Review. Prehosp. Emerg. Care.

[10] Mercer C.B., Ball M., Cash R.E., Rivard M.K., Chrzan K., Panchal A.R. (2021). Ultrasound Use in the Prehospital Setting for Trauma: A Systematic Review. Prehosp. Emerg. Care.

[11] Tricco A.C., Lillie E., Zarin W., O’Brien K.K., Colquhoun H., Levac D., Moher D., Peters M.D.J., Horsley T., Weeks L. (2018). PRISMA Extension for Scoping Reviews (PRISMA-ScR): Checklist and Explanation. Ann. Intern. Med..

[12] Jagla M., Grudzien A., Starzec K., Tomasik T., Zasada M., Kwinta P. (2019). Lung ultrasound in the diagnosis of neonatal respiratory failure prior to patient transport. J. Clin. Ultrasound.

[13] Carmo K.B., Evans N., Kluckow M., Berry A. (2013). Neonatal Ultrasound in Transport. Curr. Pediatr. Rev..

[14] Ollier V., Loi B., Rivaud C., Fortas F., Ruetsch V., Yousef N., Jourdain G., De Luca D. (2022). Semi-quantitative lung ultrasound score during ground transportation of outborn neonates with respiratory failure. Eur. J. Pediatr..

[15] Browning Carmo K., Lutz T., Berry A., Kluckow M., Evans N. (2016). Feasibility and utility of portable ultrasound during retrieval of sick term and late preterm infants. Acta Paediatr..

[16] Becerra-Hervás J., Solé-Ribalta A., Girona-Alarcón M., Campos R., Fresán E., Millán N., Alejandre C., Esteban E. (2025). Lung Ultrasound in Pediatric and Neonatal Pre-Hospital Care: An Observational Study. J. Ultrasound Med..

[17] Campos L.S., Valdovinos L.R., Fradera O.O., Montolio L.G., Rodríguez J.G. (2021). Clinical ultrasound in pediatric and neonatal interfacility transport. An. Pediatr..

[18] Mommers L., Slagt C., Rn F.C., van der Crabben R., Moors X., Dos Reis Miranda D. (2023). Feasibility of HEMS performed prehospital extracorporeal-cardiopulmonary resuscitation in paediatric cardiac arrests; two case reports. Scand. J. Trauma. Resusc. Emerg. Med..

[19] Reid C., Burns B., Gourlay S. (2023). Prehospital Ultrasound-Guided Pediatric Fascia Iliaca Block. Air Med. J..

[20] Byhahn C., Bingold T.M., Zwissler B., Maier M., Walcher F. (2008). Prehospital ultrasound detects pericardial tamponade in a pregnant victim of stabbing assault. Resuscitation.

[21] Campo dell’ Orto M., Kratz T., Wild C., Horstmann C., Walcher F., Seibel A., Hamm C., Breitkreutz R. (2014). Pre-hospital ultrasound detects pericardial tamponade in young patients with occult blunt trauma: Time for preparation? Case report and review of literature. Clin. Res. Cardiol..

[22] Steiger H.V., Rimbach K., Müller E., Breitkreutz R. (2009). Focused emergency echocardiography: Lifesaving tool for a 14-year-old girl suffering out-of-hospital pulseless electrical activity arrest because of cardiac tamponade. Eur. J. Emerg. Med..

[23] Polk J.D., Merlino J.I., Kovach B.L., Mancuso C., Fallon W.F. (2004). Fetal evaluation for transport by ultrasound performed by air medical teams: A case series. Air Med. J..

[24] Nielsen V.M.L., Bruun N.H., Sovso M.B., Klojgård T.A., Lossius H.M., Bender L., Mikkelsen S., Tarpgaard M., Petersen J.A.K., Christensen E.F. (2022). Pediatric Emergencies in Helicopter Emergency Medical Services: A National Population-Based Cohort From Denmark. Ann. Emerg. Med..

[25] Karfunkle B., Chan H.K., Fisher B., Gill J., Bakunas C., Gordon R., Miller S., Huebinger R. (2024). Prehospital Ultrasound: Nationwide Incidence from the NEMSIS Database. Prehosp. Emerg. Care.

[26] Leviter J., Auerbach M., Amick M., O’Marr J., Battipaglia T., Amendola C., Riera A. (2022). Point-of-Care Ultrasound Curriculum for Endotracheal Tube Confirmation for Pediatric Critical Care Transport Team Through Remote Learning and Teleguidance. Air Med. J..

[27] Stritzke A., Soraisham A., Murthy P., Kowal D., Paul R., Kamaluddeen M., Mohammad K., Al Awad E.H., Thomas S. (2019). Neonatal Transport Clinician Performed Ultrasound Evaluation of Cardiac Function. Air Med. J..

[28] Job S., Griksaitis M.J., Singh Y. (2023). Role of Point of Care Ultrasound in the Transport Setting for Evaluating Infants and Children with Shock. Point-of-Care Ultrasound for the Neonatal and Pediatric Intensivist: A Practical Guide on the Use of POCUS.

[29] Foster B., Kuttab H.I., Damewood S.C., Brazelton T., Al-Subu A.M. (2021). Use of Point-of-Care Ultrasound in the Pediatric and Neonatal Emergency Transport Realm. Pediatr. Ann..

[30] Browning Carmo K. (2017). The History of Ultrasound and Its Use at Point of Care: Neonatal Ultrasound in Transport. Curr. Treat. Options Pediatr..

[31] Boet A., Jourdain G., Demontoux S., Hascoet S., Tissieres P., Rucker-Martin C., De Luca D. (2017). Basic Hemodynamic Monitoring Using Ultrasound or Electrical Cardiometry During Transportation of Neonates and Infants. Pediatr. Crit. Care Med..

