# Supplementary material for: Current Practices and Gaps in Integrating Point-of-Care Ultrasound in Neonatal and Pediatric Transport: A Scoping Review
Source: Diagnostics (Basel). 2026 Feb 3;16(3):471. doi: 10.3390/diagnostics16030471 (PMC12896864; doi:10.3390/diagnostics16030471)
Supplement: Supplementary file 1 [file diagnostics-16-00471-s001.zip › File S2.pdf]

File S2: Search Criteria

Database	Search Strategy
PubMed	("Emergency Medical Services"[MeSH] OR "Ambulances"[MeSH] OR "Air Ambulances"[MeSH] OR "Transportation of Patients"[MeSH] OR prehospital[Title/Abstract] OR EMS[Title/Abstract] OR paramedic[Title/Abstract] OR ambulance*[Title/Abstract] OR air medical[Title/Abstract] OR aeromedical[Title/Abstract] OR "critical care transport"[Title/Abstract]) AND ("Point of care ultrasound"[MeSH] OR "Ultrasonography"[MeSH] OR "Point-of-Care Systems"[MeSH] OR "Echocardiography"[MeSH] OR ultrasound[Title/Abstract] OR POCUS[Title/Abstract] OR sonography[Title/Abstract] OR sonographic[Title/Abstract] OR ultrasonography[Title/Abstract] OR echocardiography[Title/Abstract]) AND ("Infant"[MeSH] OR "Child"[MeSH] OR "Adolescent"[MeSH] OR "Pediatrics"[MeSH] OR neonat[Title/Abstract] OR newborn[Title/Abstract] OR infant[Title/Abstract] OR child[Title/Abstract] OR pediatric[Title/Abstract] OR paediatric[Title/Abstract] OR adolescent[Title/Abstract] OR baby[Title/Abstract] OR babies[Title/Abstract] OR preterm[Title/Abstract] OR premature[Title/Abstract])
Scopus	(INDEXTERMS("Emergency Medical Services") OR INDEXTERMS(Ambulances) OR INDEXTERMS("Air Ambulances") OR INDEXTERMS("Transportation of Patients") OR TITLE-ABS(prehospital) OR TITLE-ABS(EMS) OR TITLE-ABS(paramedic) OR TITLE-ABS(ambulance*) OR TITLE-ABS("air medical") OR TITLE-ABS(aeromedical) OR TITLE-ABS("critical care transport")) AND (INDEXTERMS(Point of care ultrasound) OR INDEXTERMS(Ultrasonography) OR INDEXTERMS("Point-of-Care Systems") OR INDEXTERMS(Echocardiography) OR TITLE-ABS(ultrasound) OR TITLE-ABS(POCUS) OR TITLE-ABS(sonography) OR TITLE-ABS(sonographic) OR TITLE-ABS(ultrasonography) OR TITLE-ABS(echocardiography))<br>AND (INDEXTERMS(Infant) OR INDEXTERMS(Child) OR INDEXTERMS(Adolescent) OR INDEXTERMS(Pediatrics) OR TITLE-ABS(neonat) OR TITLE-ABS(newborn) OR TITLE-ABS(infant) OR TITLE-ABS(child) OR TITLE-ABS(pediatric) OR TITLE-ABS(paediatric) OR TITLE-ABS(adolescent) OR TITLE-ABS(baby) OR TITLE-ABS(babies) OR TITLE-ABS(preterm) OR TITLE-ABS(premature))
EMBASE	('emergency medical service'/exp OR 'ambulance'/exp OR 'air ambulance'/exp OR 'patient transport'/exp OR prehospital:ti,ab OR ems:ti,ab OR paramedic:ti,ab OR ambulance*:ti,ab OR air medical:ti,ab OR aeromedical:ti,ab OR 'critical care transport':ti,ab) AND ('Point of care ultrasound'/exp OR 'ultrasonography'/exp OR 'point of care system'/exp OR 'echocardiography'/exp OR ultrasound:ti,ab OR pocus:ti,ab OR sonography:ti,ab OR sonographic:ti,ab OR ultrasonography:ti,ab OR echocardiography:ti,ab) AND ('infant'/exp OR 'child'/exp OR

	'adolescent'/exp OR 'pediatrics'/exp OR neonat:ti,ab OR newborn:ti,ab OR infant:ti,ab OR child:ti,ab OR pediatric:ti,ab OR paediatric:ti,ab OR adolescent:ti,ab OR baby:ti,ab OR babies:ti,ab OR preterm:ti,ab OR premature:ti,ab)
CINAHL	(MH "Emergency Medical Services+" OR MH "Ambulances+" OR MH "Air Ambulances" OR MH "Transportation of Patients+" OR TI(prehospital OR EMS OR paramedic OR ambulance* OR "air medical" OR aeromedical OR "critical care transport") OR AB(prehospital OR EMS OR paramedic OR ambulance* OR "air medical" OR aeromedical OR "critical care transport")<br>AND (MH "Point-of-Care Systems+" OR MH "Ultrasonography+" OR MH "Echocardiography+" OR TI("point of care ultrasound" OR ultrasound OR POCUS OR sonography OR sonographic OR ultrasonography OR echocardiography) OR AB("point of care ultrasound" OR ultrasound OR POCUS OR sonography OR sonographic OR ultrasonography OR echocardiography)) AND (MH "Infant+" OR MH "Child+" OR MH "Adolescence+" OR MH "Pediatrics+" OR TI(neonat* OR newborn OR infant OR child OR pediatric OR paediatric OR adolescent OR baby OR babies OR preterm OR premature) OR AB(neonat* OR newborn OR infant OR child OR pediatric OR paediatric OR adolescent OR baby OR babies OR preterm OR premature))
Web of Science	TS=(("emergency medical services" OR "ambulances" OR "air ambulances" OR "critical care transport" OR "transportation of patients" OR prehospital OR "emergency medical services" OR paramedic OR ambulance OR "air medical" OR aeromedical) AND ("point of care ultrasound" OR "ultrasonography" OR "point-of-care systems" OR "echocardiography" OR ultrasound OR "point-of-care ultrasound" OR sonography OR sonographic OR ultrasonography OR echocardiography) AND ("infant" OR "child" OR "adolescent" OR "pediatrics" OR neonate OR newborn OR infant OR child OR pediatric OR paediatric OR adolescent OR baby OR babies OR preterm OR premature))
