# Supplementary material for: Current Practices and Gaps in Integrating Point-of-Care Ultrasound in Neonatal and Pediatric Transport: A Scoping Review
Source: Diagnostics (Basel). 2026 Feb 3;16(3):471. doi: 10.3390/diagnostics16030471 (PMC12896864; doi:10.3390/diagnostics16030471)
Supplement: Supplementary file 1 [file diagnostics-16-00471-s001.zip › File S3.pdf]

File S3: Standardized data extraction form

1. Title
2. Author(s)
3. Journal
4. Year
5. Study Design
6. Country/Region
7. Transport Context (Ground/Air, Prehospital/Interfacility)
8. Sample size
9. Patient Population (Neonatal/Pediatric, Age Range)
10. Level of Care (Tertiary/Community/Regional Transport service)
11. Indication for Transport
12. Indication for POCUS (Diagnostic, Procedural, Both)
13. POCUS Acquisition (Lung, Cardiac, Abdominal, Vascular, Procedural, Head)
14. Procedural guidance type (Vascular access / Thoracentesis / Intubation / Other)
15. Image Acquisition success rate (If available)
16. Outcomes:
    a. Management Change?
    b. Diagnostic Accuracy
    c. Procedural Success rate
    d. Safety/Adverse Events
17. Operator profession (RN, EMT, AEMT, Physician, NP/PA/APP, Fellow, Resident, Student)
18. Training level (Formal, on the job)
19. Equipment type (handheld, portable cart, other)
20. Barriers (Technological, Training, Transport, Legal)
21. Technological barriers (Equipment size, image quality, battery, transmission issues)
22. Training (lack of training, poor acquisition)
23. Transport challenges (Space, motion artifacts, lighting, patient instability)
24. Protocol used? (SAFE-R, RUSH, FAST, other)
25. Quality assurance mentioned. (Yes/No)
26. Limitations Identified by Authors
27. Gaps Identified (standardization, research needs, cost analysis, patient outcomes)
28. Study focus (Feasibility/Pilot, Descriptive, Clinical outcomes, Evaluate sensitivity/specificity, Training/Education, Telecommunication)
