# Supplementary material for: Current Practices and Gaps in Integrating Point-of-Care Ultrasound in Neonatal and Pediatric Transport: A Scoping Review
Source: Diagnostics (Basel). 2026 Feb 3;16(3):471. doi: 10.3390/diagnostics16030471 (PMC12896864; doi:10.3390/diagnostics16030471)
Supplement: Supplementary file 1 [file diagnostics-16-00471-s001.zip › File S4.pdf]

File S4: Summary of age reporting across included studies. Pediatric age groups include patients aged 1 month to 17 years. Neonatal groups include patients less than 1 month old. The "all ages" group includes neonatal, pediatric, and adult patient populations.

Reference #	Age Summary (as reported)	Type of Statistic	Age groups
[17]	50 newborns	Count, category	Neonatal
[18]	4 infants: 10 min, 4 hr, 15 min, 15 min old	Individual ages	Neonatal
[28]	60 <1 yr, 189 1-2 yr, 284 3-7 yr, 218 8-15 yr	Counts by age group	Pediatric
[29]	3291 patients which included 168 pediatric patients: 168 patients aged 0-18 years	Count, range	All ages
[22]	One 7-year-old	Single value	Pediatric
[23]	One 7-year-old	Single value	Pediatric
[24]	One 17-year-old	Single value	Pediatric
[25]	One 17-year-old	Single value	Pediatric
[26]	One 14-year-old	Single value	Pediatric
[27]	One 13-year-old	Single value	Pediatric
[36]	30 infants, mean age 29 days (SD 41)	Mean, SD	Neonatal/pediatric
[21]	23 patients, median age 85 days (range 4 hr to 6 yr)	Median, range	Neonatal/pediatric
[19]	76 newborns, mean age 3 hr (range 1.5 to 4.9 hr)	Mean, range	Neonatal
[20]	55 patients, median age 4 hr 10 min (range 47 min to 13 days)	Median, range	Neonatal
[10]	56 neonates; 33 pediatric, avg age 23.2 mo (range 14.9–81.5 mo)	Count, mean, range	Neonatal/pediatric
