# Supplementary material for: Current Practices and Gaps in Integrating Point-of-Care Ultrasound in Neonatal and Pediatric Transport: A Scoping Review
Source: Diagnostics (Basel). 2026 Feb 3;16(3):471. doi: 10.3390/diagnostics16030471 (PMC12896864; doi:10.3390/diagnostics16030471)
Supplement: Supplementary file 1 [file diagnostics-16-00471-s001.zip › File S5.pdf]

File S5. Extracted data of reported changes in clinical management or decision-making attributable to POCUS during neonatal and pediatric transport across 15 included studies.

**References (Number of pts, *n*)**	**Pts with a change in management, *n* (%)**	**Category of reported change in management**	**Number of events, *n***	**Description**
Nielsen (*n*=75)	0	None reported	0	No changes reported
Karfunkle (*n*=168)	0	None reported	0	No changes reported
Mommers (*n*=1)	1 (100%)	Resuscitation decision<br>No change	1<br>0	Focused ultrasound identified cardiac standstill and informed decision to perform cannulation for E-CPR
Byhahn (*n*=1)	1 (100%)	Procedural intervention<br>Medical management<br>Triage change<br>No change	1<br>1<br>1<br>0	Identification of cardiac tamponade led to immediate pericardial puncture, restricted fluids, and early notification to receiving hospital; therefore, a surgical team was ready upon arrival
Campo dell'Orto (*n*=1)	1 (100%)	Procedural intervention<br>Triage change<br>No change	1<br>1<br>0	Identification of cardiac tamponade led to early pericardial puncture, and early surgical team mobilization.
Steiger (*n*=1)	1 (100%)	Procedural intervention	1	Pericardial effusion was identified and drained prior to other resuscitation efforts.
Polk (*n*=1)	1 (100%)	Diagnostic clarification<br>Triage change<br>No change	1<br>1<br>0	POCUS altered transport planning as a breech fetal presentation was identified in an adolescent patient and was thus diverted to an OB-capable center.
Boet (*n*=30)	0	None reported	**0**	No changes reported
Reid (*n*=1)	1 (100%)	Medical management<br>No change	1<br>0	POCUS facilitated iliac fascial nerve block placement for pain control.
Carmo (*n*=4)	4 (100%)	Diagnostic clarification<br>Medical management<br>Triage change<br>No change	4<br>4<br>2<br>0	Each case had a change in management. Diagnosis was confirmed in all 4 cases, which led to changes in management in all 4 cases. 2/4 cases were redirected to

				cardiology centers after diagnosis of congenital heart defects.
Jagla (*n*=50)	21 (42%)	Procedural intervention<br>Airway management<br>Ventilator adjustment<br>Medical management<br>Diagnostic clarification<br>No change	3<br>6<br>7<br>5<br>0<br>29	LUS changed the course of clinical management in 21/50 (42%) neonates during their stabilization for transport, including: Decompression of PTX (n = 3 [6%]), ETT adjustment (n = 6 [12%]), ventilator setting adjustment (n = 7 [14%]), and surfactant administration (n = 5 [10%]).
Campos (*n*=23)	14 (54%)	Procedural intervention<br>Ventilator adjustment<br>Medical management<br>Diagnostic clarification<br>No change	3<br>3<br>3<br>4<br>9	In 3/23 patients, decision to drain a pneumothorax was made after POCUS. In 4/23 patients, ultrasound led to changes in medical management of volume status. In 3/23 patients, ultrasound results led to changes in ventilator settings. Ultrasound provided diagnostic clarity in 4/23 patients. Ultrasound resulted in no change in management after confirmatory results in 9/23 patients.
Ollier (*n*=76)	0	None reported	0	No changes reported
Browning-Carmo (*n*=55)	29 (52.7%)	Diagnostic clarification<br>Medical management<br>Triage change<br>No change	23<br>1<br>6<br>0	In 6/55 babies, the receiving hospital was changed based on ultrasound findings. In 5/55 babies, ultrasound confirmed normal anatomy. In 5/55 babies, ultrasound diagnosed congenital heart disease, in 1/55 babies, ultrasound found polycystic kidney disease and in 1/55 babies ultrasound confirmed diagnosis and led to change in hemodynamic support. In 11/55 babies, ultrasound discovered hemodynamic compromise.

Becerra Hervas (*n*=89)	32 (35.9%)	Ventilator adjustment<br>Triage change<br>Diagnostic clarification<br>Medical management<br>No change	14<br>3<br>4<br>11<br>57	In 14/89 (15.7%) patients, ventilation was adjusted after ruling out complications; in 3/89 (3.4%) patients, there was a change in the receiving center; in 4/89 (4.5%) patients, the diagnosis was revised; in 11/89 (12.4%) patients, fluid management was optimized.
Totals	106 (18.4%)	Diagnostic clarification<br>Resuscitation decisions<br>Medical management<br>Ventilator adjustments<br>Procedural interventions<br>Airway management<br>Transport destination<br><br>Total events	36<br>1<br>26<br>24<br>9<br>6<br>14<br><br>116	

Abbreviation: pts, patients.
One patient may have had more than one POCUS-related management change. Therefore, subtype counts do not sum to the overall number of patients who experienced a change in management.
