# Supplementary material for: Current Practices and Gaps in Integrating Point-of-Care Ultrasound in Neonatal and Pediatric Transport: A Scoping Review
Source: Diagnostics (Basel). 2026 Feb 3;16(3):471. doi: 10.3390/diagnostics16030471 (PMC12896864; doi:10.3390/diagnostics16030471)
Supplement: Supplementary file 1 [file diagnostics-16-00471-s001.zip › File S6.pdf]

File S6. Reported barriers and challenges to POCUS implementation during pediatric and neonatal transport among 15 studies with extractable patient-level data, categorized by barrier type and subtype.

	(*n*)	Reference
**Reported barriers**		
**Technological**		
Image quality	0	
Equipment size	1	Browning-Carmo
Battery issues	0	
Time to obtain ultrasound	1	Steiger
Documentation	0	
**Training**		
Lack of training	3	Campos; Ollier; Browning-Carmo
Poor image acquisition	0	
**Transport related**		
Space limitations	0	
Patient instability	0	
**Not reported**	11	Nielsen; Karfunkle; Mommers; Byhahn; Campo dell' Orto; Polk; Boet; Reid; Carmo; Jagla; Becerra Hervas
